# LncRNA ILF3AS1, MMP3, and MMP9 as well as miRNA-212 as emerging novel biomarkers for childhood epilepsy

**DOI:** 10.3389/fmolb.2024.1434023

**Published:** 2024-08-23

**Authors:** Amena Rezk Mohammed, Wafaa Abdelaziz Emam, Shaymaa A. Mohammed, Alshaymaa A. Abd Elalim, Eatemad Nabil Abdelhalim Mansour, Haidy Mahmoud Nasr, Aya A. Ghamry, Sabah M. Alkhawagah, Doaa Sadek Ahmed Fathy, Rasha Sobhy Elattar, Yasser Gaber Ibrahim Abish, Abdullah Hussein, Boshra Ahmed Zaghloul, Marwa K. Khairallah, Norah Alharbi, Salwa Seif Eldin, Amal Fahmy Dawood, Marwa A. Sabet, Marwa G. Gamea, Suzan Eid Elshishtawy Ibrahim, Aliaa A. Mosa, Marwa A. Dahpy

**Affiliations:** ^1^ Biochemistry Department, Faculty of Medicine (for Girls), Al-Azhar University, Cairo, Egypt; ^2^ Clinical Pathology Department, Faculty of Medicine (for Girls), Al-Azhar University, Cairo, Egypt; ^3^ Pediatrics Department, Faculty of Medicine (for Girls), Al-Azhar University, Cairo, Egypt; ^4^ Medical Microbiology and Immunology Department, Faculty of Medicine (for Girls), Al-Azhar University, Cairo, Egypt; ^5^ Community and Occupational Medicine Department, Faculty of Medicine (for Girls), Al-Azhar University, Cairo, Egypt; ^6^ Neurology Department, Faculty of Medicine (for Girls), Al-Azhar University, Cairo, Egypt; ^7^ Radiodiagnosis Department, Faculty of Medicine, Al-Azhar University, Cairo, Egypt; ^8^ Radiodiagnosis and Intervention Radiology Department, Faculty of Medicine (for Girls), Al-Azhar University, Cairo, Egypt; ^9^ Department of Internal Medicine, Faculty of Medicine, Assiut University, Asyut, Egypt; ^10^ Internal Medicine Department, College of Medicine, Princess Nourah Bint Abdulrahman University, Riyadh, Saudi Arabia; ^11^ Department of Basic Medical Sciences, College of Medicine, Princess Nourah Bint Abdulrahman University, Riyadh, Saudi Arabia; ^12^ Department of Microbiology and Immunology, Faculty of Pharmacy, Sphinx University, New Assiut, Egypt; ^13^ Department of Pharmacology, Faculty of Medicine, Assiut University, Asyut, Egypt; ^14^ Clinical Pathology Department, Faculty of Medicine, Ain Shams University, Cairo, Egypt; ^15^ Medical Biochemistry and Molecular Biology Department, Faculty of Medicine, Assiut University, Asyut, Egypt; ^16^ Department of Medical Biochemistry and Molecular Biology, Armed Forces College of Medicine (AFCM), Cairo, Egypt

**Keywords:** epilepsy, lncRNA ILF3AS1, miRNA-212, MMP3, MMP9, magnetic resonance imaging

## Abstract

**Background:**

Globally, approximately 70 million people suffer from epilepsy. Infants constitute a significant percentage of these cases. Hence, there is a significant need for better understanding of the pathophysiology of epilepsy through laboratory and radiological methods for early detection and optimized management. Interleukin enhancer binding factor 3 antisense RNA l (ILF3AS1) is a long non-coding RNA (lncRNA) that enhances the expressions of matrix metalloproteinase 3 (MMP3) and matrix metalloproteinase 9 (MMP9), which are considered to be epileptogenic.

**Aim:**

We aimed to assess the serum expressions of the lncRNAs ILF3AS1, MMP3, and MMP9 along with microRNA-212 (miRNA-212) as predictive biomarkers in children with epilepsy; we also assessed their correlations with magnetic resonance imaging (MRI) findings.

**Subjects and Methods:**

Fifty children with epilepsy and fifty healthy controls were considered in this study. Serum expressions of the lncRNA ILF3AS1 and miRNA-212 were estimated by quantitative real-time polymerase chain reaction (qPCR). Serum concentrations of MMP3 and MMP9 were estimated by enzyme-linked immunosorbent assay (ELISA) in parallel with MRI findings and different baseline biochemical parameters of all the subjects.

**Results:**

The results showed significantly higher levels of lncRNAs ILF3AS1, MMP3, and MMP9 as well as lower levels of miRNA-212 in children with epilepsy compared to the controls. The fold-change of miRNA-212 was a significant negative predictor (odds ratio = 0.153, *p* = 0.000). The receiver operating characteristic curves (Roc) showed that the areas under the curves for MMP3, MMP9, and lncRNA ILF3AS1 as well as the fold-change for miRNA-212 were 0.659, 0.738, 0.656, and 0.965, respectively. Brain lesions were detected in 15 patients (30%) with epilepsy, whereas the remaining 35 patients (70%) had normal results.

**Conclusion:**

Serum levels of the lncRNA ILF3AS1 among children with epilepsy were higher than those in the control group and were associated with upregulation of both MMP3 and MMP9 as well as downregulation of miRNA-212 expressions, suggesting their predictive utility in monitoring the development of epilepsy; this also means that a treatment plan focusing on the ILF3AS1/miRNA-212/MMP3/MMP9 axis could be an effective strategy for treating epilepsy.

## Introduction

Epilepsy accounts for a sizable percentage of the neurological disorders reported around the world (Zeng et al., 2022). Every year, between 50 and 70 million people are affected globally, and 2.4 million new people are diagnosed with epilepsy. Approximately 80% of all epilepsy cases are reported from developing countries, yet only three-quarters of those affected receive the necessary treatment (Cava et al., 2018). The risk of epilepsy is greater in younger and older age groups (Thijs et al., 2019), with recurrent seizures being a hallmark of pediatric epilepsy (Banawalikar et al., 2021).

The clinical techniques used to diagnose epilepsy at present include clinical manifestations, imaging examinations, and electroencephalography (EEG) (Fernandez-Baca Vaca and Park, 2020). Neuroimaging is important for establishing the etiology, providing prognosis, and planning appropriate care. The most effective imaging technique for identifying underlying epileptogenic foci and various causes is magnetic resonance imaging (MRI) (Vattipally and Bronen, 2004; Bernasconi et al., 2011; Wilmshurst et al., 2015).

There are multiple theories explaining the etiology of epilepsy, and one of the primary processes in epilepsy is neuroinflammation, which is an inflammatory response to epileptic convulsions (Zeng et al., 2022). Inflammation is both a cause and a result of temporal lobe epilepsy (TLE) (Wu et al., 2017). However, genetic background also plays a significant role in addition to inflammation (Liu et al., 2022).

Non-coding RNAs (ncRNAs) are not translated into functional proteins; they alter gene translation and interfere with the signaling pathways (Zeng et al., 2022). In addition, they serve as epigenetic factors that both participate in and are dysregulated during epileptogenesis. Abnormal ncRNA expressions have been observed in patients with and animal models of epilepsy. Thus, they may be used as biomarkers for the diagnosis of epilepsy and treatment response prognosis (Manna et al., 2022).

Non-coding RNAs exceeding 200 nucleotides in length are referred to as long non-coding RNAs (lncRNAs) that are thought to contribute to inflammation. LncRNAs serve as competing endogenous RNAs (ceRNAs) that negatively regulate microRNA (miRNA) expressions (Lekka and Hall, 2018) in the form of small ncRNAs that are 20–23 nucleotides in length and regulate gene expression (Manna et al., 2022). A newly discovered lncRNA, ILF3AS1, has been reported to be associated with dysregulated expressions in many types of cancers and epilepsy (Cai et al., 2020).

Several miRNAs are specifically expressed in the brain. Therefore, the circulating brain-enriched miRNAs are valuable sources for diagnosing pathological brain development. Many brain pathologies associated with epileptogenesis are thought to be caused by dysregulated miRNAs, including synaptic plasticity and neuroinflammation (Heiskanen et al., 2023).

Alterations in miRNA expressions may be involved in the pathogenesis of epilepsy by regulating the expressions of inflammatory factors, such as IL-1, INF-α, and tumor necrosis factor alpha (TNF-α). Of note, increased expressions of the reactive astrocytes were observed in the hippocampal sclerosis specimens of TLE patients (Wang and Zhao, 2021).

In the central nervous system (CNS), miRNA-212 has a potent modulatory effect (Brennan and Henshall, 2020); it has been linked to neurological disorders (Herrera-Espejo et al., 2019), and epilepsy-inducing circumstances, such as status epilepticus, rapidly stimulate its expression (Brennan and Henshall, 2020).

As TLE is caused by inflammation, several inflammatory agents have been detected in the brain tissues of patients suffering from refractory epilepsy. Accordingly, data suggest that increased production or activity of matrix metalloproteinase (MMP), an inflammatory mediator, after an insult may influence epileptogenesis (Cai et al., 2020).

Matrix metalloproteinase 9 (MMP9) acts on the inflammatory processes at various levels; it facilitates leukocyte influx via the blood–brain barrier (BBB) and releases chemokines from the extracellular matrix (ECM) to produce a chemotactic signal and stimulate TNF-α (Van Lint and Libert, 2007; Rivera et al., 2010; De Bock et al., 2015).

MMP3 and MMP9 expressions are known to be promoted by ILF3AS1 overexpression, leading to the hypothesis that MMP inhibitors could be reliable treatments for epilepsy. However, the currently used MMP inhibitors are non-specific, with many negative side effects. MMPs themselves may not be suitable for focused treatment as molecules upstream of the MMPs, such as ILF3AS1 (Cai et al., 2020).

Multiple studies have explored the associations between miRNA*-*212 and epilepsy, but very few studies have explored the association between the lncRNA ILF3AS1 and epilepsy, considering that miRNA-212 is targeted by lncRNA ILF3AS1, causing boomback effects on MMP3 and MMP9. Therefore, the current study was aimed at assessing the interactions between the lncRNA ILF3AS1, MMP3, MMP9, and miRNA-212 in children with epilepsy and healthy children to explore their roles as potential biomarkers and independent risk factors of epilepsy; these results were further correlated with the MRI features among children with epilepsy to better understand the pathogenesis of epilepsy and predict potential targeted therapies.

## Patients and methods

### Study design and setting

Fifty children with epilepsy and fifty healthy controls of both sexes, aged 6 months to 18 years, were included in this case–control study. The subjects were recruited from the Al Zahraa University Hospital in Cairo, Egypt, between December 2022 and May 2023.

### Ethical statement

The study protocols were reviewed and approved by the Research Ethics Committee of the Faculty of Medicine (for Girls) at Al-Azhar University (Approval No. 2022101560). Before starting the study, the parents of all the participants signed an informed consent form after clarifying the purpose of the study and ensuring its safety and confidentiality.

### Sampling technique

The sample size was calculated using the online epi info program by considering the confidence level (95%), power of the test (80%), and ratio of controls to cases (1:1), with an estimated odds ratio (OR) of 3 for detection; therefore, the sample size was calculated as 50 cases along with 50 controls. Samples were collected from the hospital departments on randomly selected days (Dean et al., 2024).

### Inclusion criteria

Children with epilepsy were recruited from the Pediatric and Neurology departments. These patients were clinically diagnosed in accordance with the International League Against Epilepsy (ILAE) criteria (Dean et al., 2024), and only patients with no family history of epilepsy, no concurrent systemic disorders, no psychiatric or neurological diseases, and normal neurological examination results were included in the study. A comparable number of age- and sex-matched apparently healthy children were recruited from the Pediatric and Neurology outpatient clinics based on selection after a routine checkup. The healthy controls had no history of epilepsy or any other neurological diseases.

### Exclusion criteria

Patients were excluded if they had had clinical seizures during the 3 days prior to blood drawing, severe neurological or neuroimmunological illnesses (such as meningitis or encephalitis), a concurrent inflammatory disease, cancer, received immunomodulatory or immunosuppressive drugs during the prior 6 months, surgery or serious trauma within the previous 2 weeks, a severe mental disease, or renal or hepatic insufficiency. In addition, patients with absolute contraindications for MRI examination, such as cardiac resynchronization therapy (CRT) devices, cardiac implantable electronic devices (CIEDs), and implantable cardioverter defibrillators (ICDs), were not included. Lastly, patients with contraindications to iodinated intravenous contrast agent administration were also excluded from this study.

## Procedures and assessments

### Full history

Information was gathered from each subject, including their age, sex, duration of epilepsy, frequency of attacks, type of seizures, time of last attack, and types of antiepileptic drugs used, to exclude pseudointractability.

### Radiological examination

Using the non-focal and temporal lobe epilepsy protocol, a 1.5 T superconducting MR imager (Achieva, Philips Medical System) was used for the scanning process.

MRI scan: Before entering the scanning room, the patient or their parents signed an approved written consent form after being appraised of the procedures.

Positioning for epilepsy MRI: Headfirst supine, with pillows under the legs for added comfort; the head was placed in a head coil for immobilization.

MRI scan localizer: A three-plane localizer was initially used to locate and design the sequences. These localizers were T1-weighted, low-resolution scans generally lasting less than 25 s (Wang et al., 2020).

T2 turbo spin echo (TSE) axial, T2 fluid-attenuated inversion recovery (FLAIR) axial, and diffusion-weighted imaging (DWI)/apparent diffusion coefficient (ADC) axial: The block was positioned parallel to the genu and splenium of the corpus callosum, and axial slices were planned along the sagittal plane. T2 TSE axial and T2 FLAIR parameters: repetition time (TR) of 4000–5500 ms, echo time (TE) of 100–120 ms, slice thickness of 1 mm, flip angle of 130°–150°, phase R > L, matrix of 320 × 320, field of view (FOV) of 210°–230°, gap between slices of 10%, average number of excitations (NEXs) of 2. DWI parameters: TR of 7000–9000 ms, TE of 70–115 ms, flip angle of 130°, average NEXs of 1–2, slice thickness of 1 mm, matrix of 192 × 192, FOV of 210°–230°, phase R > L, gap between slices of 10%, and B value of 0–1000.

T1 spin echo (SE) coronal: The positioning block was tilted perpendicular to the line extending along the genu and splenium of the corpus callosum, and coronal slices were planned along the sagittal plane. T1 parameters: TR of 500–700 ms, TE of 15–25 ms, slice thickness of 3 mm, flip angle of 90°, phase R > L, matrix of 304 × 304, FOV of 210°–230°, gap between slices of 10%, and NEXs (average) of 2.

T2 TSE sagittal: The block was positioned parallel to the midline of the brain, and sagittal slices were planned along the axial plane. T2 TSE sagittal parameters: TR of 4500–6000 ms, TE of 100–120 ms, slice thickness of 2 mm, flip angle of 130°–150°, phase A > P, matrix of 320 × 304, FOV of 210°–230°, gap between slices of 10%, and NEXs (average) of 2.

T1 and T2 TSE coronal oblique 1 mm (epilepsy protocol): The block was tilted perpendicular to the long axis of the hippocampal region, and high-resolution coronal slices were planned along the sagittal plane. T1 parameters: TR of 400–600 ms, TE of 15–25 ms, slice thickness of 1 mm, flip angle of 140°, phase R > L, matrix of 320 × 320, FOV of 170°–190°, gap between slices of 10%, NEXs (average) of 4. T2 parameters: TR of 3000–4000 ms, TE of 100–120 ms, slice thickness of 1 mm, flip angle of 130°–150°, phase A > P, matrix of 170 × 190, FOV of 170°–190°, gap between slices of 10%, and NEXs (average) of 4 (Wang et al., 2020).

T1-weighted contrast + axial: The block was positioned parallel to the genu and splenium of the corpus callosum, with axial slices planned along the sagittal plane. A contrast agent of 0.2 mL/kg (0.1 mmol/kg) was administered intravenously at 2 mL/s, for a maximum dose of 20 mL (Nardone et al., 2014).

Susceptibility-weighted imaging (SWI) or T2* axial: The block was positioned parallel to the genu and splenium of the corpus callosum, with axial slices planned along the sagittal plane.

### Laboratory investigations

Serum levels of MMP3 and MMP9 were determined using enzyme-linked immunosorbent assay (ELISA) (Cat nos. E-EL-H1446 and E0936Hu, respectively, Shanghai Korain Biotech Co.) according to the manufacturer instructions. Routine laboratory investigation include a complete blood picture, ALT, AST, serum creatinine, blood urea, serum Calcium (Ca), Pottassium (K), Sodium (Na), and Phosphorus (P) were done for all patients and controls.

## Expression levels of lncRNA ILF3AS1 and miRNA-212 using quantitative real-time polymerase chain reaction (q-PCR)

The total RNA was isolated from the whole blood samples of all groups using Direct-zol RNA Miniprep Plus (Cat no. R2072, Zymo Research Corp., United States), after which both the quantity and quality were evaluated using a Beckman dual spectrophotometer (United States). The Super Script IV One-Step real-time PCR kit (Cat no. 12594100, Thermo Fisher Scientific, Waltham, MA, United States) was used to reverse transcribe the extracted RNA, followed by q-PCR using a 96-well-plate step one instrument (Applied Biosystems, United States). The primers used for lncRNA ILF3AS1, miRNA*-*212, GAPDH, and U6 were as follows.

LncRNA ILF3AS1: forward 5′-TAAACCCCACTGTCTTCC-3′ and reverse 5′-TTCCTTGCTCTTCTTGCTC-3′; GAPDH: forward 5′-AATGGACAACTGGTCGTGGAC-3′ and reverse 5′-CCCTCCAGGGGATCTGTTTG-3′; miRNA-212: forward 5′- CCTAAGGTGGCGAGAAAATG-3′ and reverse 5′- TGCTGGCTGATACCTTCAGA-3′; U6: forward 5′-AGTAAGCCCTTGCTGTCAGTG-3′ and reverse 5′-CCTGGGTCTGATAATGCTGGG-3′. Using the 2^−∆∆Cq^ approach, the miRNA expression levels were measured and normalized to the internal reference genes, namely U6 and GAPDH.

The cyclic conditions used in the q-PCR system were as follows: initial denaturation for 40 cycles each of 10 s at 98°C, 10 s at 55°C, and 30 s at 72°C for the amplification step.

The data were expressed as cycle thresholds (Ct) for the target and housekeeping genes, with normalization to account for the variations in the expressions of each of the target genes; lncRNA ILF3AS1 and miRNA*-*212 were investigated for the mean critical threshold expressions of the GAPDH and U6 housekeeping genes, respectively, using the ΔΔCt method (Gene Copoeia, Inc. United States). Furthermore, the relative quantification of each target gene was achieved using the 2^−ΔΔCT^ method.

## Data management and statistical analyses

Statistical analyses were performed using the Statistical Package for Social Sciences (SPSS) version 22 (IBM Corp., release 2013, IBM SPSS Statistics for Windows, Version 22.0, Armonk, NY, United States). The mean ± standard deviation (SD) values were used to present the numerical data, whereas frequencies (N) and percentages (%) were used to express the categorical variables. The mean rank variations of the two independent groups were compared using the Mann–Whitney U test.

We assured the significance of our results to control the family-wise error rate across the estimated parameters tested by applying Benforoni correction that was carried out by dividing the significance level by the number of tests performed for pairwise comparisons, and the adjusted ɑ were assessed, then we compared each p-value by the ɑ values.

The area under the curve (AUC) values of the receiver operating characteristic (ROC) curves were utilized to assess the diagnostic potentials of the molecular biomarkers and metalloproteinases in the identification of epilepsy. A *p*-value of less than 0.05 was deemed significant for probability.

## Results

In this study, the mean ages of the case and control subjects were 9.4 ± 3.7 and 8 ± 4.5 years, respectively, with no statistically significant difference. The demographics and biochemical parameters related to the enrolled children are shown in Table 1.

**FIGURE 1:**
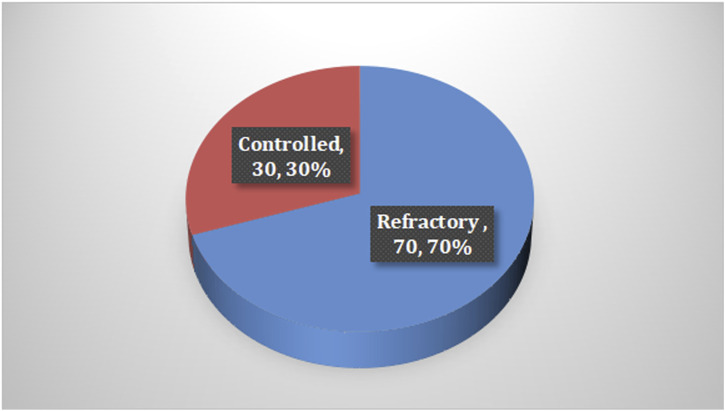
Frequency of seizures among the epileptic children.

Among the cases, it was found that 70% of children with epilepsy had been suffering for less than 5 years, and the majority of them received levetiracetam and valproic acid as monotherapy. In terms of seizures, 84% of the cases reported having generalized seizures and 70% experienced refractory seizures, with the last attack occurring less than 6 months prior to the study, as shown in Table 2 and Figure 1.

**TABLE 1:** Demographics and biochemical parameters between children with epilepsy and healthy children.

Groups Lab. profile	Cases (N = 50)	Controls (N = 50)	*p*-value
	Mean/Median ± SD	Median ± SD	
Age (years)	9.4 ± 3.8	7.9 ± 4.5	0.088
Sex Male	27 (54%)	27 (54%)	1.00
Female	23 (46%)	23 (46%)	
Duration of epilepsy - < 5 years	35 (70%)	—	—
- ≥ 5 years	15 (30%)	—	
Type of seizures - Generalized seizures	41 (82%)	—	—
- Focal seizures	9 (18%)	—	
ALT (IU/L)	13 (5.25)	19 (16.5)	0.000*
AST (IU/L)	23 (11.25)	24.5 (14.5)	0.942
Urea (mg/dL)	22 (13)	21 (11.25)	0.121
Creatinine (mg/dL)	0.4 (0.15)	0.3 (0.19)	0.022*
Ca (mg/dL)	9.7 (0.8)	9.8 (1.4)	0.029*
P (mg/dL)	4.9 (0.8)	5.1 (1.0)	0.22
Na (mmol/L)	140 (9)	142 (7.5)	0.27
K (mmol/L)	50 (0.52)	4.4 (1.03)	0.000*
RBCs **(**million cells/cmm)	84.5	78	0.068
Hemoglobin (g/dL)	11.5 (1.75)	10.9 (1.3)	0.002*
TLC ( $10^{3}$ /L)	7.5 (2.4)	6.3 (3.9)	0.058
Lymphocytes	2.9 (1.23)	2.8. (0.8)	0.967
Neutrophils	3.7 (0.13)	3.5 (2.9)	0.152
Basophils	0.5 (0.5)	0.25 (0.4)	0.015*
Platelets ( $10^{3}$ /L)	269.5 (146)	362.5 (90)	0.000*

Data are expressed as mean/median ± SD or numbers as a percent. The independent sample *t*-test was utilized for comparison between quantitative data, while chi-squared values were used for testing the significance between qualitative data. The Mann–Whitney test was used for non-parametric data. *Statistically significant difference.

**TABLE 2:** Medical history of the participating children with epilepsy.

Items	N = 50	%
o Duration of epilepsy		
- < 5 years	35	70.0
- ≥ 5 years	15	30.0
o Prescribed treatment		
- Monotherapy	47	94.0
- Combined therapy	3	6.0
o Type of seizures		
- Generalized seizures	42	84.0
- Focal seizures	8	16.0

Data are expressed as numbers and percentage (%).

**FIGURE 2:**
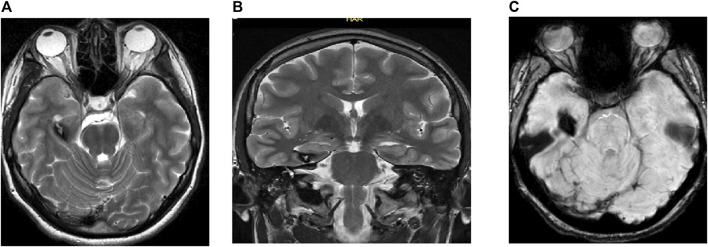
MRI of the brain of a 17-year-old man showing **(A)** axial T2 weighted image (WI), **(B)** coronal T2 WI, and **(C)** gradient echo (GRE) T2*/SWI: Figure show RT hippocampal popcorn lesion with peripheral signal loss due to hemosiderin deposition and prominent blooming in the GRE T2*/SWI image, characteristic of hippocampal cavernoma.

**FIGURE 3:**
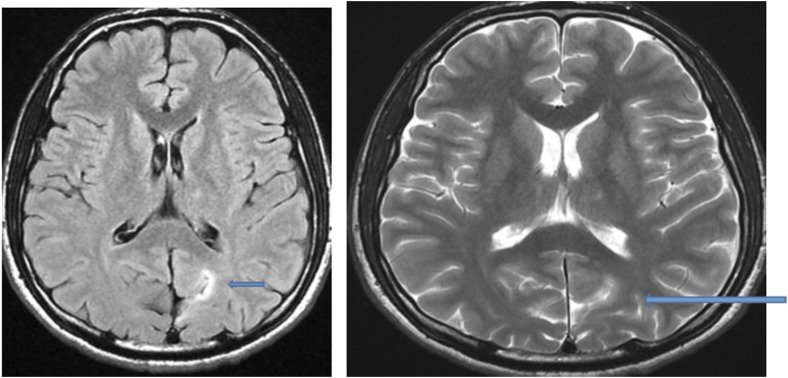
MRI of the brain of a 13-year-old male child: Axial FLAIR and T2-weighted image showing hyperintense focal thickening at the left occipitoparietal sulcus (arrow) compatible with focal cortical dysplasia.

**FIGURE 4:**
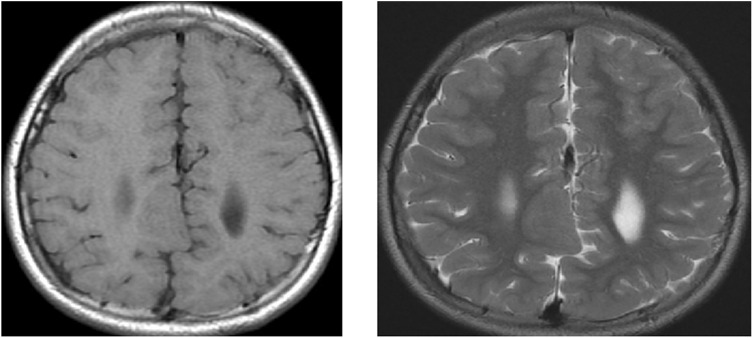
MRI of the brain of a 9-year-old child showing axial T1 weighted image (WI) and axial T2 WI for a large well-defined nodule at the RT subcortical parafalcine region abutting the falx another similar lesion seen at the left frontal region, with both lesions having isointense signals to the gray matter suggestive of gray matter heterotopia.

Using MRI as the primitive diagnostic tool for epilepsy, it was found that 30% of the cases had lesioned epilepsy, out of which mesial temporal sclerosis (MTS) represented approximately half of the cases, as shown in Table 3 and Figures 2–4.

Higher levels of MMP3 and MMP9 were found in the cases compared to controls. Furthermore, higher serum expression levels of lncRNA ILF3AS1 and lower levels of miRNA-212 were found among the cases compared to controls, with statistically significant differences, as shown in Table 4. Among the children with epilepsy, higher levels of MMP9, lncRNA ILF3AS1, and fold-change miRNA-212 were noted among those with lesions detected by MRI than those with normal findings, with no statistically significant differences, as shown in Table 5. Using logistic regression analysis, the serum expression of miRNA-212 alone was detected as a negative predictor of epilepsy with statistical significance, as shown in Table 6.

**TABLE 3:** MRI findings among the children with epilepsy.

Items	N = 50	%
Normal finding	35	70.0
Lesioned epilepsy	15	30.0
Finding of lesioned epilepsy (N = 15)		
Mesial temporal sclerosis (MTS)	7	46.7
Focal cortical dysplasia (FCD)	2	13.3
Cavernoma	2	13.3
Gray matter heterotopia	2	13.3
Pachygyria	1	6.7
Polymicrogyria	1	6.7

Data are expressed as numbers and percentage (%).

**TABLE 4:** Comparison between children with epilepsy and healthy controls regarding serum metalloproteinases and molecular biomarkers.

Group biomarkers	Cases (N = 50)	Controls (N = 50)	*p*-value
	Mean rank	Mean rank	
MMP3 (ng/mL)	58.46	42.54	0.006^*^
MMP9 (ng/L)	62.48	38.62	0.000^*^
LncRNA ILF3AS1	58.30	42.70	0.007^*^
miRNA-212 expression	27.24	73.76	0.000^*^

Data are expressed as means. The independent sample *t*-test was utilized for comparison between the quantitative data. The Mann–Whitney test was used for non-parametric data. *Statistically significant difference. MMP3, metalloproteinase 3; ILF3AS1, interleukin enhancer binding factor 3 antisense RNA l.

**TABLE 5:** Assessment of metalloproteinases and molecular biomarkers among children with epilepsy based on MRI findings.

Groups biomarkers	Lesioned positive MRI findings (N = 15)	Normal MRI findings (N = 35)	*p*-value
	Mean rank	Mean rank	
MMP3 (ng/mL)	22.23	26.9	0.3
MMP9 (ng/L)	25.7	25.4	0.949
LncRNA ILF3AS1	27.3	24.7	0.575
miRNA-212 expression	26.3	25.2	0.799

Data are expressed as means. The independent sample *t*-test was utilized for comparison between the quantitative data. The Mann–Whitney test was used for non-parametric data. *Statistically significant difference. MMP3, metalloproteinase 3; ILF3AS1, interleukin enhancer binding factor 3 antisense RNA l.

**TABLE 6:** Logistic regression for detecting epilepsy predictors among the participating children.

	B Coefficient	Sig	OR	95% confidence interval for OR	
				Lower	Upper
MMP3 (ng/mL)	−0.644	0.638	0.525	0.036	7.717
MMP9 (ng/L)	0.025	0.088	1.025	0.996	1.054
LncRNA ILF3AS1	0.591	0.198	1.806	0.734	4.444
miRNA-212 expression	−1.878	0.000*	0.153	0.067	0.350
Constant	−0.043	0.970	0.958		

*Statistically significant predictor. OR, odds ratio.

**FIGURE 5:**
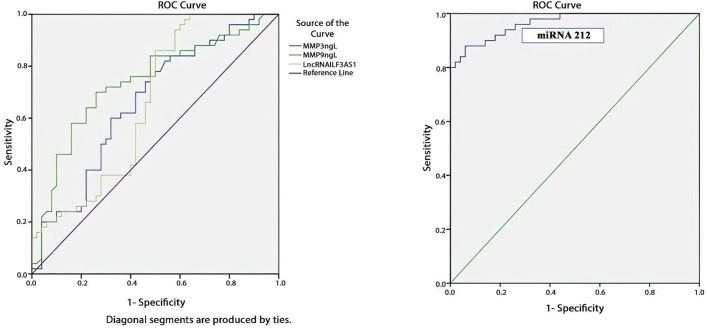
ROC curve showing that fold-change miRNA-212 has the most diagnostic power for detecting epilepsy based on the highest AUC of 0.965, as shown in Table 6.

Table 7 and Figure 5 present the evaluations of the metalloproteinases and molecular biomarkers as diagnostic tools for epilepsy based on the ROC curve. The estimated AUC values were 0.659, 0.738, 0.656, and 0.965 for MMP3, MMP9, lncRNA ILF3AS1, and fold-change miRNA-212, respectively, which are good results for MMP9 and excellent for miRNA-212.

**TABLE 7:** ROC curve data for assessment of diagnostic power of metalloproteinases and molecular biomarkers in detecting epilepsy.

Biomarkers	Cut-off point	Sensitivity (%)	Specificity (%)	AUC	*p*-value
MMP3 (ng/mL)	≥1.17	74	54	0.659	0.006^*^
MMP9 (ng/L)	≥81.5	84	52	0.738	0.000^*^
ILF3AS1 expression	≥0.30179	76	52	0.656	0.007^*^
miRNA-212 expression	≤1.99756	92	82	0.965	0.000^*^

ROC, receiver operating characteristic, AUC, area under the curve; *statistically significant result.

## Discussion

Epilepsy is one of the most prevalent neurological disorders worldwide, affecting almost 50 million individuals of various ages. However, when epilepsy is properly diagnosed and treated, up to 70% of the affected population may be able to live seizure-free (World Health Organization, 2024).

In the present study, 30% of the studied children had suffered from epilepsy for more than 5 years; of these, 84% had suffered from generalized seizures. Furthermore, 76% of the children with epilepsy had refractory-type seizures, with the last episode of seizure being registered within the last 6 months before the study; this is fairly consistent with the findings reported by El-Tallawy et al. (2013) and Khedr et al. (2013), who revealed that generalized tonic–clonic seizures were the most common type of epilepsy. In other studies, focal onset seizures were the most prevalent seizure type (Alshahawy et al., 2018; Ullah et al., 2018).

In this study, MRI was performed to correlate the radiological findings with the cause of epilepsy, where 30% of the patient group had lesioned epilepsy; however, normal findings were obtained in 70% of the cases. These findings differ only slightly from those reported by Kreilkamp et al. (2019), who noted lesioned epilepsy in 33% and normal findings in 67% of the cases.

MTS was the most common type of lesions (46.7%) in our study. However, this is not compatible with the findings of Apolot et al. (2022), who reported acquired structural brain abnormalities (61.22%) as the most common type of lesions in their study.

Apolot et al. (2022) reported hippocampal sclerosis (HS) as the most common structural abnormality, which is consistent with our finding, as noted in 46.7% of the cases.

Numerous genome-wide studies have provided evidence that ncRNA molecules play significant roles in large-scale epigenetic alterations that occur as epilepsy progresses; these are highly expressed in the brain and contribute to the control of physiological and pathophysiological processes, such as immune system activation, oxidative stress, synaptic plasticity, neuronal development, apoptosis, and neurogenesis, all of which are linked to epilepsy (Cai and Wan, 2018; Yao et al., 2019; Manna et al., 2022).

A growing number of studies have shown that epigenetic regulation involving histone modification, DNA methylation, and ncRNAs are implicated in epilepsy (Butler-Ryan and Wood, 2021).

The potential involvement of lncRNAs in epileptogenesis has been suggested based on the altered numbers and diversity of lncRNAs detected in patients with epilepsy, making them appropriate targets for treatment (Liu et al., 2022). Additionally, lncRNAs can control these processes by competitively suppressing miRNAs that are directly implicated in these functions (Ratti et al., 2020).

The aberrant production of miRNAs, which regulate protein levels by binding to their target mRNAs, is assumed to be associated with inflammatory pathways, cell death, neuronal excitability, and synaptic reorganization, which are also closely related to epileptogenesis (Li et al., 2014).

Several studies have examined various miRNAs and lncRNAs as well as their roles in epileptogenesis (Manna et al., 2022), suggesting that these lncRNAs can be used as diagnostic markers or prospective therapeutic targets for epilepsy. However, the findings of these studies have not been verified in independent cohorts (Ghafouri-Fard et al., 2022).

Regarding the lab profiles in the present study, higher levels of the lncRNA ILF3AS1 were found in patients with epilepsy than the matched controls, which is consistent with the results reported by Cai et al. (2020).

Neuroinflammation, BBB dysfunction, and altered synaptic remodeling functions are the potential contributors to seizures (Wang et al., 2024).

Moreover, the serum levels of the metalloproteinases (MMP3 and MMP9) were elevated in the epilepsy group compared to the control group with significant differences.

The seizure activity triggers activation of various pathways within the CNS, including matrix metalloproteases, which result in distinct responses in the brain with the progression of necroptosis as a type of regulated cell death. These responses involve the activation of neurons and astrocytes, consequently leading to increased expressions of proteins and genes (Mohseni-Moghaddam et al., 2024).

This finding is consistent with that of the studies conducted by Broekaart et al. (2020) and Cai et al. (2020), who reported that serum levels of MMP3 and MMP9 were overexpressed in children with epilepsy, suggesting that ILF3AS1 promoted their expression. The present results are also consistent with the findings of Korotkov et al. (2018), who reported elevated MMP3 expressions in the hippocampus of TLE patients, and Rempe et al. (2018), who reported increased MMP9 activity in a TLE rat model (Broekaart et al., 2020).

Kaczmarek et al. (2023) also discussed and enforced the effects of MMP9; they stated that MMP9 is frequently generated and released in excess in reaction to stressors that have the potential to cause epileptogenesis. Moreover, the pathophysiology of epilepsy has been linked to abnormal synaptic plasticity. Increased MMP9 levels are also a result of neuroinflammation, which is another proepileptic brain response (Kaczmarek et al., 2023).

In this study, miRNA-212 was found to be downregulated, which is similar to the findings of Cai et al. (2020), who showed that the serum and hippocampal miRNA-212 levels of TLE patients decreased by 50%; this is also consistent with the findings of Haenisch et al. (2015), who screened for miRNA-212-3p in the hippocampal focal and non-focal samples of brain tissues in temporal neocortex TLE patients.

Several studies have demonstrated that lncRNAs inhibit miRNA expression (Geng et al., 2018; Han et al., 2018; Zhao et al., 2019). In our study, it was found that increased expression of lncRNA ILF3AS1 was associated with lower miRNA-212 expression in the epilepsy patients than the controls with a statistically significant difference, suggesting that lncRNA ILF3AS1 induced downregulation of miRNA-212 expression. This finding is compatible with that of Hu et al. (2019), who demonstrated that ILF3AS1 knockdown suppressed miRNA-212 expression, which in turn lowered osteosarcoma cell proliferation, invasion, and migration while provoking apoptosis.

In summary, we found that lncRNA ILF3AS1, miRNA-212, MMP3, and MMP9 have predictive and diagnostic powers for epilepsy, with AUC values of 0.656, 0.965, 0.659, and 0.738, respectively.

## Conclusion

According to our study, peripheral blood gene expression profiling provided the preliminary clues that lncRNAs ILF3AS1 and miRNA-212 are dysregulated in human epilepsy, so they may act as independent predictors of epilepsy.

The serum lncRNA ILF3AS1 expression levels were higher in children with epilepsy and were associated with higher levels of MMP3 and MMP9 as well as lower levels of miRNA-212 than those in the control group. This provides easy, sensitive, and accessible diagnostic biomarker tools for epilepsy.

Therefore, it is plausible that targeting these pathways of the ILF3AS1/miRNA-212/MMP3/MMP9 axis may potentially be a promising approach to improve the prognosis as well as manage epileptic seizures.

## Data Availability

The raw data supporting the conclusions of this article will be made available by the authors without undue reservation.

## Abbreviations

ADC, apparent diffusion coefficient; AUC, area under the curve; BBB, blood–brain barrier; ceRNAs, competing endogenous RNAs; CIED, cardiac implantable electronic device; CNS, central nervous system; CRT, cardiac resynchronization therapy; Ct, cycle threshold; DWI, diffusion-weighted imaging; ECM, extracellular matrix; EEG, electroencephalography; ELISA, enzyme-linked immunosorbent assay; FCD, focal cortical dysplasia; FLAIR, fluid-attenuated inversion recovery; FOV, field of view; GRE, gradient echo; HS, hippocampal sclerosis; ICD, implantable cardioverter defibrillator; ILF3AS1, interleukin enhancer binding factor 3 antisense RNA l; lncRNA, long non-coding RNA; miRNA-212, microRNA 212; MMP, matrix metalloproteinase; MRI, magnetic resonance imaging; MTS, mesial temporal sclerosis; ncRNA, non-coding RNA; NEX, number of excitations; OR, odds ratio; *p*-value, probability value; q-RTPCR, quantitative real-time polymerase chain reaction; ROC, receiver operator characteristic; SD, standard deviation; SE, spin echo; SWI, susceptibility-weighted imaging; TE, echo time; TLE, temporal lobe epilepsy; TNF-α, tumor necrosis factor alpha; TR, repetition time; TSE, turbo spin echo.

## References

[B1] Alshahawy A. K. Darwish A. H. Elsaid Shalaby S. Mawlana W. (2018). Prevalence of idiopathic epilepsy among school children in Gharbia Governorate, Egypt. Brain Dev. 40 (4), 278–286. 10.1016/j.braindev.2017.12.009 29295801

[B2] Apolot D. Erem G. Nassanga R. Kiggundu D. Tumusiime C. M. Teu A. (2022). Brain magnetic resonance imaging findings among children with epilepsy in two urban hospital settings, Kampala-Uganda: a descriptive study. BMC Med. Imaging 22 (1), 175. 10.1186/s12880-022-00901-7 36203127 PMC9541090

[B3] Banawalikar N. Adiga S. Adiga U. Shenoy V. Kumari S. Shetty P. (2021). Association of UGT1A6 gene polymorphism with clinical outcome in pediatric epileptic patients on sodium valproate monotherapy. Braz J. Med. Biol. Res. 54 (9), e11097. 10.1590/1414-431X2021e11097 34133540 PMC8208771

[B4] Bernasconi A. Bernasconi N. Bernhardt B. C. Schrader D. (2011). Advances in MRI for ‘cryptogenic’ epilepsies. Nat. Rev. Neurol. 7 (2), 99–108. 10.1038/nrneurol.2010.199 21243016

[B5] Brennan G. P. Henshall D. C. (2020). MicroRNAs as regulators of brain function and targets for treatment of epilepsy. Nat. Rev. Neurol. 16, 506–519. 10.1038/s41582-020-0369-8 32546757

[B6] Broekaart D. W. M. van Scheppingen J. Anink J. J. Wierts L. van Het Hof B. Jansen F. E. (2020). Increased matrix metalloproteinases expression in tuberous sclerosis complex: modulation by microRNA 146a and 147b *in vitro* . Neuropathol. Appl. Neurobiol. 46 (2), 142–159. 10.1111/nan.12572 31183875 PMC7217197

[B7] Butler-Ryan R. Wood I. C. (2021). The functions of repressor element 1-silencing transcription factor in models of epileptogenesis and post-ischemia Metab. Brain Dis. 10.1007/s11011-021-00719-2 PMC8272694 33813634

[B8] Cai X. Long L. Zeng C. Ni G. Meng Y. Guo Q. (2020). LncRNA *ILF3-AS1* mediated the occurrence of epilepsy through suppressing hippocampal miR-212 expression. Aging (Albany NY) 12 (9), 8413–8422. 10.18632/aging.103148 32404536 PMC7244033

[B9] Cai Y. Wan J. (2018). Competing endogenous RNA regulations in neurodegenerative disorders: current challenges and emerging insights. Front. Mol. Neurosci. 11, 370. 10.3389/fnmol.2018.00370 30344479 PMC6182084

[B10] Cava C. Manna I. Gambardella A. Bertoli G. Castiglioni I. (2018). Potential role of miRNAs as theranostic biomarkers of epilepsy. Mol. Ther. Nucleic Acids 13, 275–290. 10.1016/j.omtn.2018.09.008 30321815 PMC6197620

[B11] Dean A. G. Sullivan K. M. Soe M. M. (2024). OpenEpi: open-source epidemiologic Statistics for public Health, version. Available at: www.OpenEpi.com.

[B12] De Bock M. Wang N. Decrock E. Bultynck G. Leybaert L. (2015). Intracellular cleavage of the cx43 C-terminal domain by matrix-metalloproteases: a novel contributor to inflammation? Mediat. Inflamm. 2015, 257471. 10.1155/2015/257471 PMC4573893 26424967

[B13] El-Tallawy H. N. Farghaly W. M. Shehata G. A. Abdel-Hakeem N. M. Rageh T. A. Abo-Elftoh N. A. (2013). Epidemiology of epilepsy in new valley governorate, Al kharga district, Egypt. Epilepsy Res. 104 (1-2), 167–174. 10.1016/j.eplepsyres.2012.08.010 22981337

[B14] Fernandez-Baca Vaca G. Park J. T. (2020). Focal EEG abnormalities and focal ictal semiology in generalized epilepsy. Seizure 77, 7–14. 10.1016/j.seizure.2019.12.013 31882201

[B15] Geng J. F. Liu X. Zhao H. B. Fan W. F. Geng J. J. Liu X. Z. (2018). LncRNA UCA1 inhibits epilepsy and seizure-induced brain injury by regulating miR-495/Nrf2-ARE signal pathway. Int. J. Biochem. Cell Biol. 99, 133–139. 10.1016/j.biocel.2018.03.021 29608952

[B16] Ghafouri-Fard S. Hussen B. M. Jamali E. Branicki W. Taheri M. Akbari Dilmaghani N. (2022). Role of lncRNAs and circRNAs in epilepsy. Ageing Res. Rev. 82, 101749. 10.1016/j.arr.2022.101749 36216292

[B17] Haenisch S. Zhao Y. Chhibber A. Kaiboriboon K. Do L. V. Vogelgesang S. (2015). SOX11 identified by target gene evaluation of miRNAs differentially expressed in focal and non-focal brain tissue of therapy-resistant epilepsy patients. Neurobiol. Dis. 77, 127–140. 10.1016/j.nbd.2015.02.025 25766675 PMC4404495

[B18] Han C. L. Ge M. Liu Y. P. Zhao X. M. Wang K. L. Chen N. (2018). Long non-coding RNA H19 contributes to apoptosis of hippocampal neurons by inhibiting let-7b in a rat model of temporal lobe epilepsy. Cell Death Dis. 9 (6), 617. 10.1038/s41419-018-0496-y 29795132 PMC5966382

[B19] Heiskanen M. Das Gupta S. Mills J. D. van Vliet E. A. Manninen E. Ciszek R. (2023). Discovery and validation of circulating microRNAs as biomarkers for epileptogenesis after experimental traumatic brain injury-the EPITARGET cohort. Int. J. Mol. Sci. 24 (3), 2823. 10.3390/ijms24032823 36769143 PMC9918096

[B20] Herrera-Espejo S. Santos-Zorrozua B. Álvarez-González P. Lopez-Lopez E. Garcia-Orad Á. (2019). A systematic review of MicroRNA expression as biomarker of late-onset alzheimer's disease. Mol. Neurobiol. 56 (12), 8376–8391. 10.1007/s12035-019-01676-9 31240600

[B21] Hu X. H. Dai J. Shang H. L. Zhao Z. X. Hao Y. D. (2019). SP1-mediated upregulation of lncRNA *ILF3-AS1* functions a ceRNA for miR-212 to contribute to osteosarcoma progression via modulation of SOX5. Biochem. Biophys. Res. Commun. 511, 510–517. 10.1016/j.bbrc.2019.02.110 30819403

[B22] Kaczmarek K. T. Protokowicz K. &Kaczmarek L. (2023). Matrix metalloproteinase-9: a magicdrug target in neuropsychiatry? J. Neurochem. 00, 1–12. 10.1111/jnc.15976 37791997

[B23] Khedr E. M. Shawky O. A. Ahmed M. A. Elfetoh N. A. Al Attar G. Ali A. M. (2013). A community based epidemiological study of epilepsy in Assiut Governorate/Egypt. Epilepsy Res. 103 (2-3), 294–302. 10.1016/j.eplepsyres.2012.08.006 22948127

[B24] Korotkov A. Broekaart D. W. M. van Scheppingen J. Anink J. J. Baayen J. C. Idema S. (2018). Increased expression of matrix metalloproteinase 3 can be attenuated by inhibition of microRNA-155 in cultured human astrocytes. J. Neuroinflammation 15 (1), 211. 10.1186/s12974-018-1245-y 30031401 PMC6054845

[B25] Kreilkamp B. A. K. Das K. Wieshmann U. C. Biswas S. Marson A. G. Keller S. S. (2019). Neuroradiological findings in patients with “non-lesional” focal epilepsy revealed by research protocol. Clin. Radiol. 74 (1), 78.e1–78. 10.1016/j.crad.2018.08.013 30274684

[B26] Lekka E. Hall J. (2018). Noncoding RNAs in disease. FEBS Lett. 592 (17), 2884–2900. 10.1002/1873-3468.13182 29972883 PMC6174949

[B27] Li M. M. Li X. M. Zheng X. P. Yu J. T. Tan L. (2014). MicroRNAs dysregulation in epilepsy. Brain Res. 1584, 94–104. 10.1016/j.brainres.2013.09.049 24096213

[B28] Liu S. Fan M. Ma M. D. Ge J. F. Chen F. H. (2022). Long non-coding RNAs: potential therapeutic targets for epilepsy. Front. Neurosci. 16, 986874. 10.3389/fnins.2022.986874 36278003 PMC9582525

[B29] Manna I. Fortunato F. De Benedittis S. Sammarra I. Bertoli G. Labate A. (2022). Non-coding RNAs: new biomarkers and therapeutic targets for temporal lobe epilepsy. Int. J. Mol. Sci. 23 (6), 3063. 10.3390/ijms23063063 35328484 PMC8954985

[B30] Mohseni-Moghaddam P. Khaleghzadeh-Ahangar H. Atabaki R. (2024). Role of necroptosis, a regulated cell death, in seizure and epilepsy. Neurochem. Res. 49, 1–13. 10.1007/s11064-023-04010-x 37646959

[B31] Nardone B. Saddleton E. Laumann A. E. Edwards B. J. Raisch D. W. McKoy J. M. (2014). Pediatric nephrogenic systemic fibrosis is rarely reported: a RADAR report. Pediatr. Radiol. 44 (2), 173–180. 10.1007/s00247-013-2795-x 24057195 PMC3946726

[B32] Ratti M. Lampis A. Ghidini M. Salati M. Mirchev M. B. Valeri N. (2020). MicroRNAs (miRNAs) and long non-coding RNAs (lncRNAs) as new tools for cancer therapy: first steps from bench to bedside. Target Oncol. 15 (3), 261–278. 10.1007/s11523-020-00717-x 32451752 PMC7283209

[B33] Rempe R. G. Hartz A. M. S. Soldner E. L. B. Sokola B. S. Alluri S. R. Abner E. L. (2018). Matrix metalloproteinase-mediated blood-brain barrier dysfunction in epilepsy. J. Neurosci. 38 (18), 4301–4315. 10.1523/JNEUROSCI.2751-17.2018 29632167 PMC5932641

[B34] Rivera S. Khrestchatisky M. Kaczmarek L. Rosenberg G. A. Jaworski D. M. (2010). Metzincin proteases and their inhibitors: foes or friends in nervous system physiology? J. Neurosci. 30 (46), 15337–15357. 10.1523/JNEUROSCI.3467-10.2010 21084591 PMC3072038

[B35] Thijs R. D. Surges R. O’Brien T. J. Sander J. W. (2019). Epilepsy in adults. lancet 393, 689–701. 10.1016/S0140-6736(18)32596-0 30686584

[B36] Ullah S. Ali N. Khan A. Ali S. Nazish H. R. (2018). The epidemiological characteristics of epilepsy in the province of khyber pakhtunkhwa, Pakistan. Front. Neurol. 9, 845. 10.3389/fneur.2018.00845 30459698 PMC6232227

[B37] Van Lint P. Libert C. (2007). Chemokine and cytokine processing by matrix metalloproteinases and its effect on leukocyte migration and inflammation. J. Leukoc. Biol. 82 (6), 1375–1381. 10.1189/jlb.0607338 17709402

[B38] Vattipally V. R. Bronen R. A. (2004). MR imaging of epilepsy: strategies for successful interpretation. Neuroimaging Clin. N. Am. 14 (3), 349–372. 10.1016/j.nic.2004.04.002 15324853

[B39] Wang I. Bernasconi A. Bernhardt B. Blumenfeld H. Cendes F. Chinvarun Y. (2020). MRI essentials in epileptology: a review from the ILAE Imaging Taskforce. Epileptic Disord. 22 (4), 421–437. 10.1684/epd.2020.1174 32763869

[B40] Wang J. Zhao J. (2021). MicroRNA dysregulation in epilepsy: from pathogenetic involvement to diagnostic biomarker and therapeutic agent development. Front. Mol. Neurosci. 14, 650372. 10.3389/fnmol.2021.650372 33776649 PMC7994516

[B41] Wang Q. Lin Z. Yao C. Liu J. Chen J. Diao L. (2024). Meta-analysis of MMP-9 levels in the serum of patients with epilepsy. Front. Neurosci. 18, 1296876. 10.3389/fnins.2024.1296876 38449733 PMC10914997

[B42] Wilmshurst J. M. Gaillard W. D. Vinayan K. P. Tsuchida T. N. Plouin P. Van Bogaert P. (2015). Summary of recommendations for the management of infantile seizures: task force report for the ILAE commission of pediatrics. Epilepsia 56 (8), 1185–1197. 10.1111/epi.13057 26122601

[B43] World Health Organization (2024). Epilepsy. Available at: https://www.who.int/en/news-room/fact-sheets/detail/epilepsy.

[B44] Wu Z. Zhou L. Ding G. Cao L. (2017). Overexpressions of miR-212 are associated with poor prognosis of patients with pancreatic ductal adenocarcinoma. Cancer Biomark. 18 (1), 35–39. 10.3233/CBM-160671 27814273 PMC13020612

[B45] Yao R. W. Wang Y. Chen L. L. (2019). Cellular functions of long noncoding RNAs. Nat. Cell Biol. 21 (5), 542–551. 10.1038/s41556-019-0311-8 31048766

[B46] Zeng C. Hu J. Chen F. Huang T. Zhang L. (2022). The coordination of mTOR signaling and non-coding RNA in regulating epileptic neuroinflammation. Front. Immunol. 13, 924642. 10.3389/fimmu.2022.924642 35898503 PMC9310657

[B47] Zhao T. Ding Y. Li M. Zhou C. Lin W. (2019). Silencing lncRNA PVT1 inhibits activation of astrocytes and increases BDNF expression in hippocampus tissues of rats with epilepsy by downregulating the Wnt signaling pathway. J. Cell Physiol. 234 (9), 16054–16067. 10.1002/jcp.28264 30805931

